# Evolutionary Patterns and Selective Pressures of Odorant/Pheromone Receptor Gene Families in Teleost Fishes

**DOI:** 10.1371/journal.pone.0004083

**Published:** 2008-12-31

**Authors:** Yasuyuki Hashiguchi, Yoshimi Furuta, Mutsumi Nishida

**Affiliations:** Division of Molecular Marine Biology, Ocean Research Institute, University of Tokyo, Tokyo, Japan; University of Western Cape, South Africa

## Abstract

**Background:**

Teleost fishes do not have a vomeronasal organ (VNO), and their vomeronasal receptors (V1Rs, V2Rs) are expressed in the main olfactory epithelium (MOE), as are odorant receptors (ORs) and trace amine-associated receptors (TAARs). In this study, to obtain insights into the functional distinction among the four chemosensory receptor families in teleost fishes, their evolutionary patterns were examined in zebrafish, medaka, stickleback, fugu, and spotted green pufferfish.

**Methodology/Principal Findings:**

Phylogenetic analysis revealed that many lineage-specific gene gains and losses occurred in the teleost fish TAARs, whereas only a few gene gains and losses have taken place in the teleost fish vomeronasal receptors. In addition, synonymous and nonsynonymous nucleotide substitution rate ratios (K_A_/K_S_) in TAARs tended to be higher than those in ORs and V2Rs.

**Conclusions/Significance:**

Frequent gene gains/losses and high K_A_/K_S_ in teleost TAARs suggest that receptors in this family are used for detecting some species-specific chemicals such as pheromones. Conversely, conserved repertoires of V1R and V2R families in teleost fishes may imply that receptors in these families perceive common odorants for teleosts, such as amino acids. Teleost ORs showed intermediate evolutionary pattern between TAARs and vomeronasal receptors. Many teleost ORs seem to be used for common odorants, but some ORs may have evolved to recognize lineage-specific odors.

## Introduction

Olfaction is a sense for recognizing chemicals in the external environment. In many animals, olfaction is essential for various activities such as foraging, migration, and reproduction. Most terrestrial vertebrates have two distinct chemosensory organs, the main olfactory epithelium (MOE) and the vomeronasal organ (VNO). Generally, the MOE is considered to recognize environmental chemicals, while the VNO recognizes pheromones, although recent studies have reported some exceptions [1], [2]. It has been known that a distinct set of chemosensory receptors is expressed in each organ. In the MOE, main odorant receptors (ORs) and trace amine-associated receptors (TAARs) are expressed, whereas in the VNO, vomeronasal receptors type 1 (V1Rs) and type 2 (V2Rs) are expressed [reviewed in 3]. This implies that in terrestrial vertebrates, ORs and TAARs are mainly used to recognize “ordinary” odorants, whereas V1Rs and V2Rs are used to recognize pheromones. Indeed, several mammalian V1Rs and V2Rs respond to pheromone candidates [4]–[6].

Unlike the terrestrial vertebrates, teleost fishes do not have a VNO and their V1Rs and V2Rs are expressed in the MOE, as are ORs and TAARs [7], [8]. In teleost fishes, functional studies of the chemosensory receptors have not been reported. Thus, in teleost fishes, it is not clear which family of chemosensory receptors is used for detecting environmental chemicals or pheromones. Also, it remains unknown whether the different types of teleost fish chemosensory receptors respond to different classes of odorants. However, recent electrophysiological studies have indirectly suggested putative ligands of each type of odorant/pheromone receptors. For instance, goldfish sex pheromones 17α, 20β-dihydroxy-4-pregnen-3-one (17, 20-P) and F-prostaglandins are suggested to be perceived by ORs [9], whereas amino acids (major environmental odorants for teleost fishes) are considered to be detected by both ORs and V2Rs [9], [10].

The copy number, pattern of diversification, and selective constraints of a chemosensory receptor gene family seem to reflect the biological functions of receptors encoded to the gene family. For example, a chemosensory receptor family that perceives species-specific pheromones may show large variation across species. In contrast, if receptors in a family are used mainly for environmental odorants, the repertoire of these receptors may be more similar between different species. In terrestrial vertebrates, it is suggested that chemosensory receptors expressed in MOE (ORs and TAARs) are broadly tuned generalists, whereas vomeronasal receptors (V1Rs and V2Rs) are narrowly tuned specialists. In mammals, chicken, and frogs, this “differential tuning hypothesis” was supported by careful comparison of the evolutionary patterns between the two types of chemosensory receptor gene families [11]. However, the evolutionary patterns of the four chemosensory receptor families have not been studied in teleost fishes.

**Figure 1:**
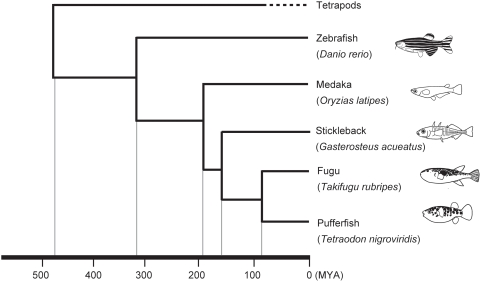
Phylogenetic relationship and estimated divergence times of the five model fishes inferred from [12].

In this study, to obtain insights into the function and biological roles of the four chemosensory receptor families in teleost fishes, we analyzed the evolutionary patterns of the four gene families by comparing the gene repertoires from zebrafish *Danio rerio*, medaka *Oryzias latipes*, stickleback *Gasterosteus aculeatus*, fugu *Takifugu rubripes*, and spotted green pufferfish *Tetraodon nigroviridis*, for which draft genome sequences are publicly available. Their phylogenetic relationship and divergence times are well studied (Fig. 1), and these data enables us more detailed comparison of the evolutionary dynamics of chemosensory receptor gene families in teleost fishes. In this paper, we analyzed evolutionary patterns of the four chemosensory receptor gene families in teleost fishes. We also discussed the similarity and differences of the evolutionary modes of chemosensory receptors between teleost fishes and tetrapods.

## Results

### Proportions of species-specific genes in the four chemosensory receptor gene families

From database searches and gene predictions, we identified 53 and 95 putatively functional (i.e. not disrupted by stop codon and/or frameshift) OR genes in medaka and stickleback, respectively. We also found 23 V2R genes in stickleback and 17 TAAR genes in pufferfish. Nucleotide sequences of these genes are available as supporting information (Texts S1, S2, S3, and S4; Data S1, S2, S3, and S4). In this study, phylogenetic trees of ORs, TAARs, V1Rs, and V2Rs in five model fishes were constructed using these sequences and published data (see Materials and methods).

**Figure 2:**
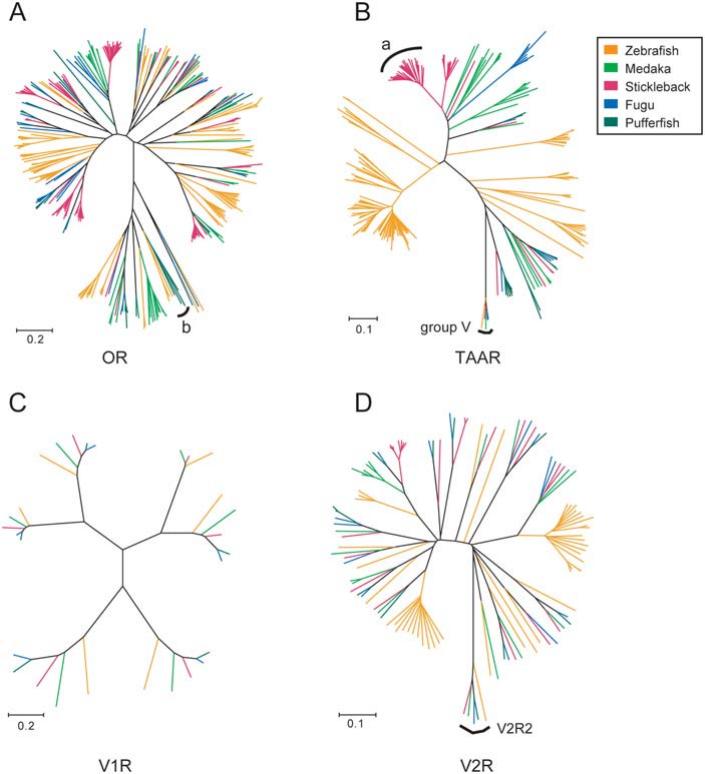
Unrooted phylogenetic trees of putatively functional (A) ORs, (B) TAARs, (C) V1Rs, and (D) V2Rs in zebrafish, medaka, stickleback, fugu, and pufferfish. The trees were reconstructed using the neighbor-joining method with Poisson-corrected protein distances. The color of each branch indicates species.

Fig. 2 shows the unrooted phylogenetic trees of ORs, TAARs, V1Rs, and V2Rs in the five fishes. Many species-specific clades were observed in teleost ORs and TAARs. In contrast, such species-specific clusters were rarely seen in teleost V2Rs. In teleost V1Rs, no species-specific gene duplications were found. To quantify the differences in phylogenetic patterns among the four evolutionary distinct chemosensory receptor families, the proportion of “species-specific” genes (i.e., genes that originated from species-specific gene gains and/or losses; see Materials and methods) was estimated for each chemosensory receptor family in each species. The numbers of chemosensory receptor genes and species-specific genes, as well as the proportion of species-specific genes in each chemosensory receptor family are shown in Table 1. For each of the five species, the proportion of species-specific genes in TAARs was higher than those of ORs and V2Rs (Table 1), although with the exception of one comparison between TAARs and V2Rs in fugu (*p* = 0.011, Fisher's exact test), the differences were not significant. With respect to the total numbers of genes in the five species, the proportion of species-specific TAAR genes was significantly higher than those of OR (*p* = 0.012) and V2R (*p* = 0.002) genes. The difference in the proportion of species-specific genes between ORs and V2Rs was not significant (*p* = 0.156). These trends might reflect the differences of the evolutionary patterns in the teleost fish chemosensory receptor families.

**Table 1:** Numbers of putatively functional genes, species-specific genes, and the proportion of species-specific genes in OR, TAAR, V1R, and V2R gene families in five fishes.

Gene family		Zebrafish	Medaka	Stickleback	Fugu	Pufferfish	Total
OR	No. of genes	102	53	95	44	42	336
	No. of species-specific genes	71	27	70	10	15	193
	Proportion of species-specific genes	0.70	0.51	0.74	0.23	0.36	0.57
TAAR	No. of genes	109	25	49	13	17	213
	No. of species-specific genes	101	14	40	8	11	174
	Proportion of species-specific genes	0.93	0.56	0.82	0.62	0.65	0.82
V1R	No. of genes	6	6	6	5	5	28
	No. of species-specific genes	0	0	0	0	0	0
	Proportion of species-specific genes	0.00	0.00	0.00	0.00	0.00	0.00
V2R	No. of genes	46	17	23	15	11	112
	No. of species-specific genes	31	6	10	0	1	48
	Proportion of species-specific genes	0.67	0.35	0.44	0.00	0.09	0.43

### Selective forces operating on each teleost chemosensory receptor gene family

**Figure 3:**
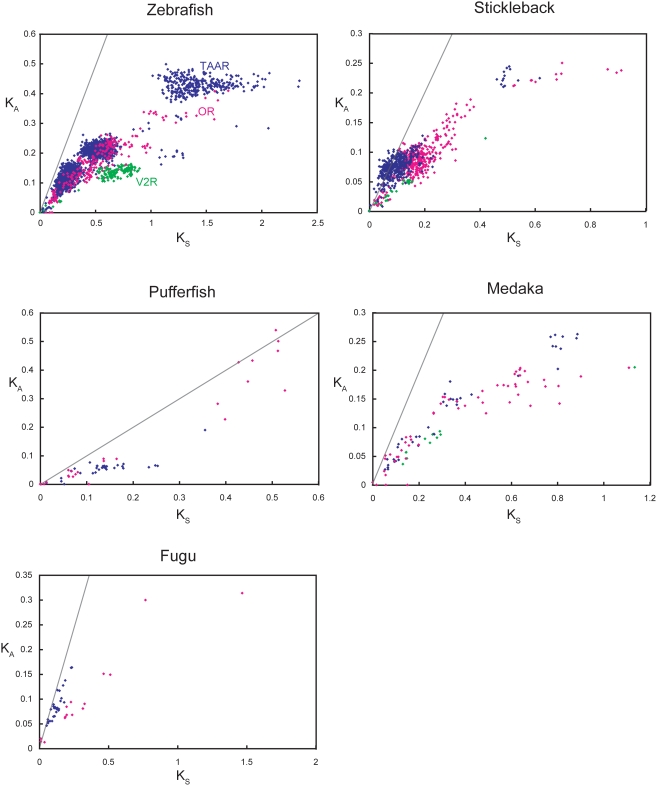
Numbers of synonymous and nonsynonymous substitutions per site of the species-specific genes in five fishes. The gray line indicates K_S_ = K_A_.

Fig. 3 shows scatterplots of pairwise non-synonymous (K_A_) and synonymous (K_S_) nucleotide substitution rates of the species-specific OR, TAAR, and V2R genes in the five fishes examined. In fugu and pufferfish, K_A_ and K_S_ values in V2Rs were not estimated because virtually no species-specific V2Rs were found. Selective constraints for each of three chemosensory receptor families were substantially different. In zebrafish, stickleback, and fugu, K_A_/K_S_ ratios of TAARs were higher than those of ORs. In particular, most stickleback TAAR genes in clade *a* (Fig. 2) showed more than 1.0 (ca. 1.0–2.1) pairwise K_A_/K_S_ ratios. This indicates that the clade *a* TAAR genes in stickleback have evolved under positive selection. Also in zebrafish and stickleback, V2R genes showed relatively lower K_A_/K_S_ ratios than OR and TAAR genes (Fig. 3). This suggests that most V2R genes in these teleosts have evolved under strong functional constraints. However, these trends were not common in all teleost fish species examined. In pufferfish, K_A_/K_S_ ratios of OR genes were higher than those of TAAR genes, which was opposite of the zebrafish, stickleback, and fugu results (Fig. 3). Interestingly, several distantly related (K_S_: 0.4–0.55) pairs of OR genes showed high K_A_/K_S_ ratios (ca. 0.7–1.0) in pufferfish. All of these genes belonged to clade *b* (Fig. 2). In contrast to the other teleost fishes, the K_A_/K_S_ ratios of species-specific genes in medaka did not clearly differ among OR, TAAR, and V2R gene families (Fig. 3).

## Discussion

### Evolutionary patterns of the four chemosensory receptor gene families in teleost fishes

In this study, we estimated the frequencies of species-specific gene gains/losses of the four evolutionary distinct chemosensory receptor gene families in zebrafish, medaka, stickleback, fugu, and pufferfish (Table 1), by calculating the proportions of species-specific genes from the phylogenetic trees (Fig. 2). We also estimated the selective constraints for each chemosensory receptor gene family (Fig. 3). In this section, we summarize the evolutionary patterns of the four chemosensory receptor gene families. Discussion in this section is somewhat speculative because there is no functional data of fish odorant/pheromone receptors.

The proportion of species-specific genes was lower in ORs than in TAARs, although some species-specific clades were observed in all species (Fig. 2). In addition, the K_A_/K_S_ ratios of OR genes in zebrafish, stickleback, and fugu were clearly lower than those of TAAR genes (Fig. 3). These characteristics imply that most teleost ORs are conserved among lineages. Thus, teleost ORs are likely to perceive “common” odorants for fishes. However, a portion of teleost ORs may have evolved to recognize some species-specific chemicals. For instance, clade *b* ORs in pufferfish (Fig. 2) showed substantially high K_A_/K_S_ ratios (Fig. 3). These ORs are considered to have evolved under positive selection. This implies that in the pufferfish lineage, the clade *b* ORs might adapt to odorants of some lineage-specific environments, such as freshwater habitats.

Phylogenetic analyses have revealed that many teleost TAARs formed species-specific clades (Fig. 2). In all species examined, the proportion of lineage-specific genes in the TAAR family was higher than those in the other families (Table 1). Exceptionally, group V TAARs [13] had strict orthologs in all teleost fish species (Fig. 2). It was suggested that these genes were not expressed in the olfactory organ [13]. Thus, the group V TAARs may have some other functions than odor detection. In zebrafish, stickleback, and fugu, K_A_/K_S_ ratios of TAAR genes were substantially higher than those of OR and V2R genes (Fig. 3). In particular, the K_A_/K_S_ ratios of many stickleback TAARs in clade *a* exceeded 1.0 (Fig. 3), indicating strong positive selection. Such a species-specific gene repertoire and high K_A_/K_S_ ratios may imply that teleost TAARs are used for detecting species-specific chemicals, such as pheromones. In mice, TAARs are known to be the receptors for biogenic amines [14]. Physiological studies have shown that teleost fishes have olfactory sensitivity to several biogenic amines [15]–[17]. Interestingly, catecholamines or their metabolites are thought to be used for chemical communication in goldfish *Carassius auratus* [15]. Odor-based mate choice and conspecific recognition have been reported in stickleback [18], [19], suggesting that pheromonal cues are crucial for their reproduction and pre-mating isolation. The clade *a* TAAR genes might be involved in the reproductive behaviors in stickleback.

In contrast to ORs and TAARs, the repertoires of vomeronasal-type odorant receptors seem to be conserved among lineages. In teleost fishes, only five or six V1R genes have been identified in each species [20]. All V1R genes retain 1∶1 orthologs, no species-specific gene duplications being observed (Table 1). This may suggest that teleost V1Rs are used for detecting odor chemicals that are important for all teleost fishes.

Phylogenetic analysis revealed that most V2R genes including V2R2 genes had strict orthologs in five fishes examined (Fig. 2). The proportion of species-specific genes in teleost V2Rs is lower than those of ORs and TAARs (Table 1). The K_A_/K_S_ ratios of V2R genes are also smaller than those of ORs and TAARs (Fig. 3). Teleost V2Rs are suggested to perceive amino acids [9], [10] that are very common environmental odors for teleost fishes [21]. In many teleost species, to recognize and to discriminate amino acids is very important for survival. Thus, the repertoire and functions of V2R genes may be maintained among teleost fish species.

### Contrasting evolutionary modes between teleost fish and tetrapod chemosensory receptor genes

Our analysis revealed that, in teleost fishes, proportions of species-specific genes were not clearly different among different receptor families (Table 1), although slight differences were suggested. This is a very contrasting evolutionary pattern to the tetrapod chemoreceptors. In tetrapods, the proportions of species-specific OR and TAAR genes are much lower than those of V1Rs and V2Rs [11]. This pattern could be explained by the emergence of the vomeronasal organ in the tetrapod lineage. Mammalian V1Rs and V2Rs are expressed exclusively in the vomeronasal organ that is considered to detect pheromones [3]. Indeed, several V1Rs and V2Rs are shown to respond to pheromonal substances [6], [22]. In tetrapods, separation of the two chemosensory organs, the VNO and the MOE, may promote functional changes of vomeronasal receptors. On the other hand, teleost V1Rs and V2Rs might maintain their original functions as receptors for environmental odors, such as amino acids.

The most remarkable difference in the evolution of chemoreceptor gene families between tetrapods and teleosts is the difference of TAAR gene repertoires. Teleost fishes have relatively larger number of TAAR genes than that in tetrapods. In addition, the proportion of species-specifc TAAR genes was higher in teleost fishes, suggesting that fish TAARs have experienced frequent gene gains/losses. In tetrapods, pheromone recognition by vomeronasal receptors may reduce the functional significance of TAARs, or may cause the use of different type of pheromonal substances, instead of biogenic amines. This might explain the relatively small TAAR gene repertoires in frogs, chicken, and mammals [23].

In this study, we showed the contrasting evolutionary patterns among ORs, TAARs, V1Rs, and V2Rs in teleost fishes. We also revealed that several OR genes in pufferfish and TAAR genes in stickleback were under positive selection. These genes are possibly involved in some lineage-specific adaptive evolutions. Our results provide useful information for future functional studies in teleost fish olfaction.

## Materials and Methods

### Data mining

The sequence data of teleost fish chemoreceptor genes were collected from the following studies: zebrafish and fugu ORs [24]; pufferfish ORs [25]; teleost V1Rs [20]; V2Rs in zebrafish, medaka, fugu, and pufferfish [25]; TAARs in zebrafish, medaka, stickleback, and fugu [13]. Second, we newly identified medaka and stickleback ORs, stickleback V2Rs, and pufferfish TAARs from their draft genome sequences, by using TBLASTN searches and profile hidden Markov Model (HMM)-based gene prediction. The detailed method of gene identification is described in [25]. The nucleotide sequences of these genes and the list of their genomic positions are available as supplementary information.

### Data analysis

For each of the four chemosensory receptor gene families, deduced amino acid sequences were aligned by the program MAFFT 5.861 [26]. Phylogenetic trees were constructed using the neighbor-joining method with Poisson-corrected protein distances implemented in the program MEGA 4 [27].

From the phylogenetic trees, we estimated the species-specific gene gains and losses by the method described in [11]. If a species-specific clade consists of *n* genes, at least *n-1* events of gene gains/losses must have taken place in the clade since the species diverged from its closest relative in the five fishes. Thus, the total proportion of genes belonging to species-specific clades is defined as the sum of these *n-1* genes for all species-specific clades divided by the total number of genes for that chemosensory receptor type in that species [11]. Species-specific clades were identified from the phylogenetic trees as clades supported with >50% bootstrap values.

To test selective constraints to the chemosensory receptors in teteost fishes, K_S_ and K_A_ values among all pairs of genes within each lineage-specific clade were calculated in OR, TAAR, and V2R gene families. The K_A_ and K_S_ values were estimated by Nei and Gojobori (1986) method [28] using DnaSP version 4.0 [29].

## Supporting Information

Text S1 Names and positions of medaka OR genes and pseudogenes.
(0.02 MB XLS)

Text S2 Names and positions of stickleback OR genes and pseudogenes.
(0.04 MB XLS)

Text S3 Names and positions of pufferfish TAAR genes and pseudogenes.
(0.03 MB XLS)

Text S4 Names and positions of stickleback V2R genes and pseudogenes.
(0.04 MB XLS)

Data S1 Nucleotide sequences of medaka OR genes and pseudogenes (FASTA format text file).
(0.06 MB TXT)

Data S2 Nucleotide sequences of stickleback OR genes and pseudogenes (FASTA format text file).
(0.11 MB TXT)

Data S3 Nucleotide sequences of pufferfish TAAR genes and pseudogenes (FASTA format text file).
(0.02 MB TXT)

Data S4 Nucleotide sequences of stickleback V2R genes and pseudogenes (FASTA format text file).
(0.07 MB TXT)
